# Optimal endoscopy timing in patients with acute variceal bleeding: A systematic review and meta-analysis

**DOI:** 10.1038/s41598-020-60866-x

**Published:** 2020-03-04

**Authors:** Da Hyun Jung, Cheal Wung Huh, Na Jin Kim, Byung-Wook Kim

**Affiliations:** 1 Department of Internal Medicine, Severance Hospital, Yonsei University College of Medicine, Seoul, Republic of Korea; 2 Division of Gastroenterology, Department of Internal Medicine, College of Medicine, Incheon St. Mary’s Hospital, The Catholic University of Korea, Seoul, Republic of Korea; 3 Medical Library, The Catholic University of Korea, College of Medicine, Seoul, Republic of Korea; 4 Present Address: Department of Internal Medicine, Yongin Severance Hospital, Yonsei University College of Medicine, Seoul, Republic of Korea

**Keywords:** Upper gastrointestinal bleeding, Liver diseases

## Abstract

Although current guidelines recommend performing endoscopy within 12 hours for acute variceal bleeding (AVB), the optimal timing remains controversial. This study aimed to assess the effect of endoscopy timing on the mortality and rebleeding rates in AVB through a systematic review and meta-analysis of all eligible studies. PubMed, Cochrane Library, and Embase were searched for relevant publications up to January 2019. Overall mortality, rebleeding rate, and other clinical outcomes were determined. For the non-randomized studies, the risk of bias assessment tool was used to assess the methodological quality of the included publications. The Mantel-Haenszel random-effects model of the RevMan software (Cochrane) and the inverse variance method were used to analyse binary end points and continuous outcomes, respectively. This meta-analysis included five studies with 854 and 453 participants who underwent urgent (≤12 hours) and non-urgent endoscopies (>12 hours), respectively. All the included studies were retrospective in nature, because of obvious ethical issues. No significant differences in the severity indexes were found between the urgent and non-urgent groups. Three studies showed 6-week mortality and the others in-hospital mortality as main outcomes. No significant difference in overall mortality rate was found between the groups (odds ratio [OR]: 0.72, 95% confidence interval [CI]: 0.36–1.45, *p* = 0.36). The rebleeding rate was similar between the two groups (OR: 1.21, 95% CI: 0.76–1.93, *p* = 0.41). Other outcomes such as successful haemostasis, need for salvage therapy, length of hospital stay, and number of blood transfusions were also similar between the groups. We demonstrated that endoscopy timing does not affect the mortality or rebleeding rate of patients with AVB. Therefore, an appropriate timing of endoscopy would be more important than an urgent endoscopy depending on each patient’s condition.

## Introduction

Acute variceal bleeding (AVB) is a common complication of cirrhosis. It is associated with a mortality rate of 20–25%^1,2^. Endoscopic therapy (e.g., band ligation and sclerotherapy) has been used as the treatment of choice for controlling AVB, in accordance with most guidelines that recommend endoscopic therapy to be performed within 12 hours of admission^3,4^. However, these recommendations are based on “expert opinion” and require corroborating evidence.

To date, several studies have been conducted on the timing of endoscopy for patients with AVB. Some studies showed that endoscopy timing is not associated with mortality or rebleeding rate in patients with AVB^5–7^. However, in two studies published in Taiwan, non-urgent endoscopy appeared to be an independent risk factor of mortality in patients with AVB^8,9^. Such inconsistency between studies raised questions about the optimal timing of endoscopy for patients with AVB. Conducting a randomized controlled trial would be the best way to determine the optimal endoscopy timing but would be difficult because of obvious ethical concerns.

Although several studies have reported the optimal endoscopy timing in patients with AVB, whether urgent endoscopy decreases composite outcomes in patients with AVB remains unclear. Therefore, the objective of this study was to conduct a meta-analysis of trials to evaluate the effect of endoscopy timing on mortality and rebleeding in patients with AVB.

## Materials and Methods

### Literature search strategy

In this study, systematic review and meta-analysis were performed on the basis of the principles of the PRISMA (Preferred Reporting Items for Systematic reviews and Meta-Analyses) statement^10^. PubMed, Cochrane Library, and Embase (from inception to January 2019) were searched independently by two authors (CWH and DHJ). The following search string was used: esophageal and gastric varices [Mesh Terms] OR gastroesophageal varices [Text Word] OR esophageal varices [Text Word] OR gastric varices [Text Word] OR hemorrhage [Mesh Terms] OR gastrointestinal hemorrhage [Mesh Terms] OR variceal bleeding [Text Word] AND endoscopy [Mesh Terms] OR esophagoscopy [Mesh Terms] OR gastroscopy [Mesh Terms] OR hemostasis [Mesh Terms] OR endoscopic band ligation [Text Word] OR endoscopic sclerotherapy [Text Word], endoscopy timing [Text Word]. The detailed search strategies in each database are shown in Supplementary Table 1. Cited references in published studies were manually and repetitively searched to identify other studies. The latest date for updating our search was January 31, 2019.

### Study selection

In the first stage of the study selection, the title and abstract of articles that our keyword search returned were scrutinized to rule out irrelevant articles. No language restriction was imposed in this stage. Next, in accordance with our inclusion and exclusion criteria, the full texts of all selected studies were screened. The inclusion criteria were as follows: (1) a diagnosis of AVB; (2) urgent endoscopic haemostasis (e.g., endoscopic band ligation and sclerotherapy) as an intervention; (3) non-urgent endoscopic haemostasis as a comparator; and (4) mortality or rebleeding as outcome. The exclusion criteria were as follows: (1) review article; (2) guidelines or consensus documents or expert position papers; (3) comments, letters, brief reports, and protocol studies; (4) case reports; (5) abstract-only publications; (6) publication in a language other than English; (7) meta-analysis articles; or (8) studies using sclerotherapy as initial treatment for oesophageal variceal bleeding.

### Data extraction

Two review authors (CWH and DHJ) independently extracted data from the included studies using a pre-data extraction form. The titles and abstracts of all included studies were reviewed to exclude irrelevant publications. Discrepancies in data interpretation were resolved by discussion, re-review of studies, and consultation with one other author (BWK) when necessary. The following information was extracted: year of publication, first author, study design, age and sex of patients, sample size, inclusion and exclusion criteria of the trial, aetiology of liver disease, Child-Pugh score, Model for End-stage Liver Disease (MELD) score, endoscopy timing, mortality rate, rebleeding rate, successful haemostasis, need for salvage therapy, length of hospital stay, and number of blood units transfused. Whereas endoscopy performed after >12 hours was considered non-urgent endoscopy, endoscopy performed in ≤12 hours was considered as urgent endoscopy. Mortality was defined as in-hospital or 6-week mortality. Rebleeding was defined as hematemesis, melena, or haematochezia with accompanying changes in laboratory findings or vital signs.

### Methodological quality

The Risk of Bias Assessment tool for Non-randomized Studies (RoBANS) was used for the assessment of the methodological quality of the included publications^11^. RoBANS has six domains. It is a validated tool that is reliable and feasible for assessing the methodological quality of non-randomized studies. Two authors (CWH and DHJ) independently assessed the methodological quality of the included studies. Any disagreement between the two evaluators was resolved by discussion.

### Statistical analysis

We used Review Manager version 5.3 (RevMan for Windows 7, the Nordic Cochrane Centre, Copenhagen, Denmark). The Mantel-Haenszel random-effect model was used for binary end points, and the inverse variance method was used for continuous outcomes. The random-effect model was chosen because it could take into account the possibility of heterogeneity between studies. The *I*^2^ test developed by Higgins was used for determining heterogeneity^12^. It measures the percentage of total variation across studies. *I*^2^ was calculated as follows: *I*^2^ (%) = 100 × (Q-df)/Q, where *Q* is Cochrane’s heterogeneity statistics and *df* was the degree of freedom. Heterogeneity was quantified on the basis of the following cut-off criteria: 0–25%, low; 25–50%, moderate; and >50%, high heterogeneity^12^. In cases of significant heterogeneity (*I*^2^ >25%), the methodological section of each study was reassessed for determining whether any discrepancy could be identified. Following this process, sensitivity analyses were performed to rule out the discrepant study. A *p* value of < 0.05 was considered statistically significant.

## Results

### Study selection

**Figure 1:**
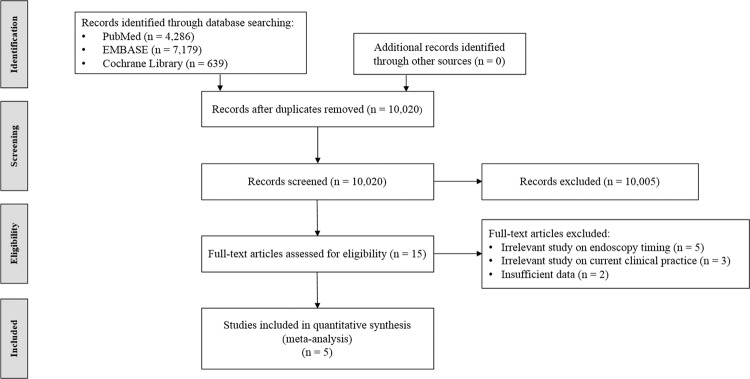
Flow diagram of the studies included in the meta-analysis.

Figure 1 summarizes the flow diagram of the selection of studies for inclusion in the meta-analysis. A total of 12,104 studies were identified in our literature search. Duplicated articles (n = 2,084) were excluded. After initial screening of titles and abstracts, 10,005 studies were additionally excluded. The full texts of the remaining 15 studies were then thoroughly reviewed. Among these studies, 10 articles were excluded from the final analysis due to the following reasons: irrelevant study on endoscopy timing (n = 5), irrelevant study on current clinical practice (n = 3), and insufficient data (n = 2). The remaining five studies were included in the final quantitative analysis.

### Characteristics of the included studies

The baseline characteristics of the included studies are listed in Table 1. A total of 1,307 patients were included, with 854 and 453 patients in the urgent and non-urgent groups, respectively. All the articles were retrospective studies published within the last 10 years (2009–2019). They were from Taiwan (n = 2), Korea (n = 2), and Canada (n = 1).

**Table 1:** Baseline characteristics of the five studies included.

Authors	Year	Study design	Patients (n)	Sex (M/F)	Age (mean), years	Aetiology of liver disease (n)	Child-Pugh score (mean)	MELD score (mean)
Cheung *et al*.^6^	2009	Retrospective	urgent: 134 non-urgent: 76	179/31	55	139 alcohol/59 viral/47 other	8.5	14.3
Hsu *et al*.^9^	2009	Retrospective	urgent: 176 non-urgent: 135	228/83	55	46 alcohol/228 viral/37 other	NA	11.6
Chen *et al*.^8^	2012	Retrospective	urgent: 54 non-urgent: 47	85/16	57	18 alcohol/74 viral/9 other	9*	13*
Yoo *et al*.^5^	2018	Retrospective	urgent: 173 non-urgent: 101	207/67	58	69 alcohol/162 viral/43 other	NA	15.9
Huh *et al*.^7^	2019	Retrospective	urgent: 317 non-urgent: 94	291/120	54	240 alcohol/136 viral/31 other	8.3	12.1

MELD, model for end-stage liver disease; NA, not available.

*Data expressed as median values.

All the studies except two (Chen study^8^ and Hsu study^9^) compared the baseline characteristics between the two arms. In all the studies except one^9^, endoscopies performed in ≤12 and >12 hours were considered urgent and non-urgent endoscopies, respectively. In the study of Hsu *et al*.^9^, urgent endoscopy was defined as an endoscopy performed within 15 hours; and non-urgent endoscopy, as endoscopy performed after 15 hours.

Except in the study by Hsu *et al*.^9^ no significant differences in the severity indexes (MELD score, Child-Pugh score, vital sign, prognostic score, and infection) were found between the urgent and non-urgent groups (Supplementary Table 2). In two studies^5,7^, the main outcomes, such as mortality and rebleeding rates, did not show significant differences between the two groups after propensity score matching. We added information related to the treatment outcome in Supplementary Table 3. All the studies included in this meta-analysis used vasoactive agents. Moreover, prophylactic antibiotics were used in all the studies except one^9^. We could not find information about prophylactic antibiotics in one study^9^. The proportion of patients with oesophageal varix bleeding was greater than that of the patients with gastric varix bleeding in the pooled analysis. All the studies included in the meta-analysis showed 6-week mortality^5,7,8^ or in-hospital mortality^6,9^ as main outcomes. The cause of death was reported in two studies^6,8^. We could check the definition of rebleeding in three studies. The definition of rebleeding in most of the studies was new-onset hematemesis, melena, or haematochezia with accompanying changes in laboratory findings (decrease in haemoglobin level by >2 g/dL within 24 hours) or vital signs (decrease in systolic blood pressure to <100 mmHg or increase in heart rate to >100 beats/min).

### Methodological quality

**Figure 2:**
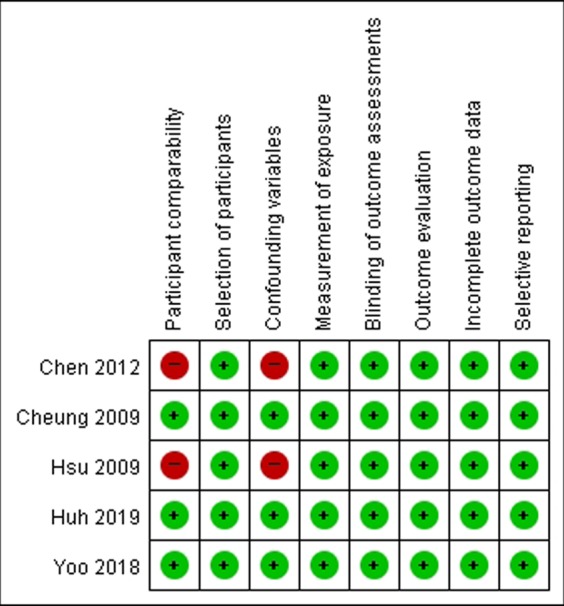
Risk of bias of the enrolled studies.

All the included studies were observational retrospective studies. The methodological qualities of the included studies were similar. Two studies had a high risk of participant comparability and confounding variables^8,9^. It did not compare baseline characteristics between the two arms. Figure 2 details the quality of these included studies.

### Outcomes

#### Mortality

**Figure 3:**
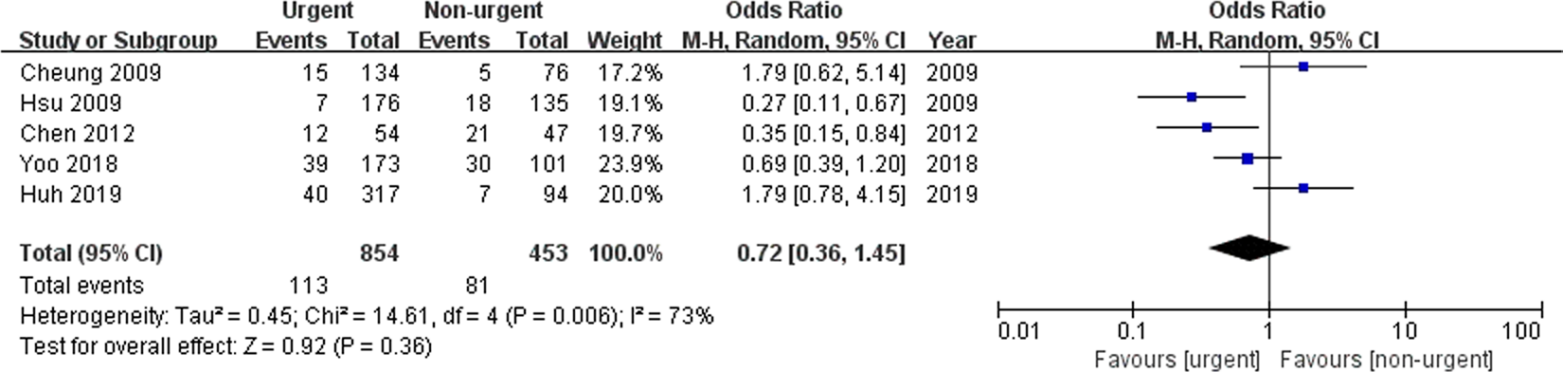
Forrest plot of the overall mortality rate for comparison between the urgent and non-urgent groups.

All the studies reported data on mortality for 1,307 patients as shown in Fig. 3. The pooled analysis revealed that the overall mortality rate was similar between the urgent and non-urgent groups (odds ratio [OR]: 0.72, 95% confidence interval [CI]: 0.36–1.45, *p* = 0.36). A high heterogeneity was observed between the studies (*p* = 0.006, *I*^2^ = 73%). A sensitivity analysis was performed after excluding studies that did not compare baseline characteristics between the two arms^8,9^. This analysis did not eliminate the heterogeneity between the studies (*p* = 0.09, *I*^2^ = 58%) (Supplementary Table 4).

#### Rebleeding

**Figure 4:**
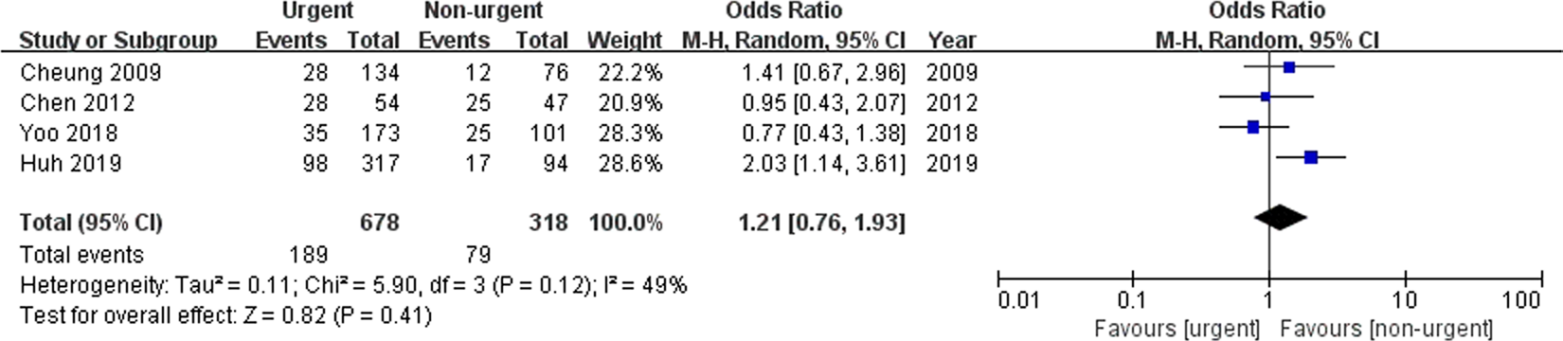
Forrest plot of rebleeding for comparison between the urgent and non-urgent groups.

Data on rebleeding were available for four studies that reported the information of 996 patients as shown in Fig. 4. The pooled analysis revealed that the rebleeding rate was similar between the urgent and non-urgent groups (OR: 1.21, 95% CI: 0.76–1.93, *p* = 0.41). A moderate heterogeneity was observed between the studies (*p* = 0.12, *I*^2^ = 49%). A sensitivity analysis was performed, excluding the study that did not compare baseline characteristics between the two arms^8^. This analysis did not eliminate the heterogeneity between the studies (*p* = 0.07, *I*^2^ = 63%) (Supplementary Table 4).

#### Other clinical outcomes

**Figure 5:**
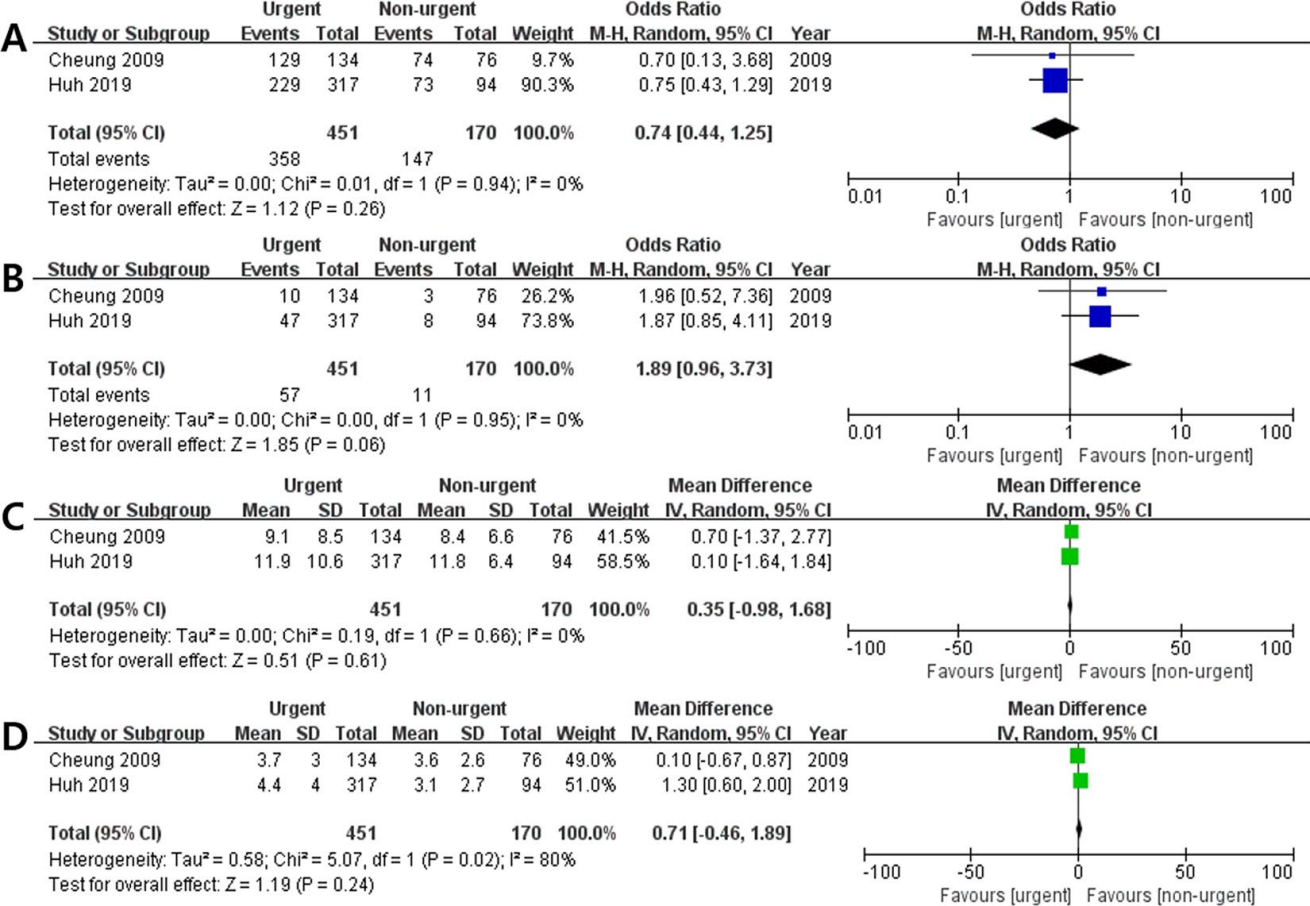
Forrest plot of other outcomes for comparison between the urgent and non-urgent groups. (**A**) Successful haemostasis; (**B**) need for salvage therapy; (**C**) length of hospital stay; (**D**) number of blood transfusions.

Table 2 summarizes the other outcomes according to endoscopy timing in the included studies. Data on other outcomes (successful haemostasis, need for salvage therapy, length of hospital stay, and number of blood transfusions) were available in two studies that reported the information of 621 patients (Fig. 5, Table 2)^6,7^. The pooled analysis demonstrated that other outcomes were similar between the two groups (Fig. 5, Table 2).

**Table 2:** Overall mortality, rebleeding, successful haemostasis, need for salvage therapy, length of hospital stay, and number of units transfused in the five studies included.

Authors	Patients (n)	Death (n)	Rebleeding (n)	Successful haemostasis (n)	Need for salvage therapy* (n)	LOS (mean), days	Number of blood transfusions (mean), units per patient
Cheung *et al*.	urgent: 134 non-urgent: 76	urgent: 15 non-urgent: 5	urgent: 28 non-urgent: 12	urgent: 129 non-urgent: 74	urgent: 10 non-urgent: 3	urgent: 9.1 non-urgent: 8.4	urgent: 3.7 non-urgent: 3.6
Hsu *et al*.	urgent: 176 non-urgent: 135	urgent: 7 non-urgent: 18	NA	254**	57**	NA	NA
Chen *et al*.	urgent: 54 non-urgent: 47	urgent: 12 non-urgent: 21	urgent: 28 non-urgent: 25	NA	NA	NA	NA
Yoo *et al*.	urgent: 173 non-urgent: 101	urgent: 39 non-urgent: 30	urgent: 35 non-urgent: 25	NA	NA	urgent: 4.0*** non-urgent: 4.0***	NA
Huh *et al*.	urgent: 317 non-urgent: 94	urgent: 40 non-urgent: 7	urgent: 98 non-urgent: 17	urgent: 229 non-urgent: 73	urgent: 47 non-urgent: 8	urgent: 11.9 non-urgent: 11.8	urgent: 4.4 non-urgent: 3.0

LOS, length of hospital stay; NA, not available.

*This outcome included balloon tamponade, additional endoscopic therapy, or transjugular intrahepatic portosystemic shunt.

**Only the total events were available.

***Data expressed as median values.

## Discussion

This systematic review and meta-analysis aimed to compare the effect of endoscopy timing on mortality and rebleeding in patients with AVB. We found no significant differences in overall mortality (OR: 0.72, 95% CI: 0.36–1.45, *p* = 0.36) or rebleeding rate (OR: 1.21, 95% CI: 0.76–1.93, *p* = 0.41) according to endoscopy timing. Other clinical outcomes were also similar between the urgent and non-urgent groups.

Although most guidelines suggest performing endoscopy within 12 hours, the optimal endoscopy timing for AVB remains controversial^3,4^. Hsu *et al*. compared urgent endoscopy with non-urgent endoscopy and showed that non-urgent endoscopy is significantly associated with increased risk of mortality^9^. However, unlike the recommendations of the guidelines, they defined urgent endoscopy as that performed within 15 hours. They did not provide data regarding baseline characteristics between the urgent and non-urgent groups either. Similarly, Chen *et al*. revealed that urgent endoscopy is associated with better outcome in patients with hematemesis^8^. However, endoscopy timing was not associated with mortality or rebleeding in patients without hematemesis. They also did not show data regarding baseline characteristics between the urgent and non-urgent groups. By contrast, Cheung *et al*. showed no significant association between time to endoscopy and mortality or rebleeding in the patients with hemodynamically stable AVB^6^. However, they included only patients who presented with hemodynamically stable variceal bleeding. More recently, in two studies published in Korea, endoscopy timing was not associated with morality or rebleeding in patients with AVB^5,7^. They included more patients than the previous studies and adjusted for various confounders by performing propensity score matching.

In the present meta-analysis, endoscopy timing did not influence mortality or rebleeding rate in the patients with AVB. One study showed that urgent endoscopy may even be harmful to some patients in terms of mortality and rebleeding^7^. The study divided patients into a low-risk group (MELD score ≤17) and a high-risk group (MELD score >17) according to the severity of the underlying liver disease. It concluded that urgent endoscopy was significantly associated with poorer outcome in low-risk patients, whereas endoscopy timing was not associated with outcome in the high-risk patients. Some possible explanations for this result are as follows: First, the quality of endoscopic examination and therapy is likely to be suboptimal in urgent endoscopy owing to the remaining food and blood in the stomach. Second, urgent endoscopy may be related with suboptimal resuscitation. It could be plausible that sufficient time for pre-endoscopic optimization of the patient’s clinical state would be more determinant than the endoscopy timing in some patients.

The results of this meta-analysis suggest that prognostic factors more crucial than endoscopy timing exist in patients with AVB. Several studies revealed that factors associated with worsened prognosis include the severity of liver disease (MELD or Child-Pugh score), shock at the time of hospital admission, infection, and hepatocellular carcinoma^13–17^. Among these factors, the severity of liver disease has been considered as a well-known prognostic factor. It is closely associated with clinical outcome such as mortality^15,18^. In patients with significant comorbid illness, stable hemodynamics, or preserved liver function, postponing endoscopic intervention until adequate medical therapy is administrated (e.g., intravenous vasopressin, antibiotics, and fluid resuscitation) could be beneficial and allow time to optimize the patients’ conditions^6,7^. Therefore, an “appropriate timing of endoscopy” would be more important than an “urgent endoscopy” depending on the patient’s condition.

Although this was the first systematic review and meta-analysis to compare the effect of endoscopy timing on mortality and rebleeding in patients with AVB, it had several limitations. First, all the included studies were retrospective in nature without randomization. This could have led to considerable selection bias in our study. As most guidelines recommend performing endoscopy within 12 hours, the number of patients in the urgent group was higher than that in the non-urgent group in this meta-analysis. In most studies, the criteria for urgent endoscopy included the endoscopist’s decision to perform urgent endoscopy in consideration of the patient’s condition and the feasibility of endoscopy. As these are subjective criteria according to the endoscopist’s decision, this might cause risk of bias. However, we could not find objective criteria for deciding between urgent and non-urgent endoscopies. In addition, the patients who underwent urgent endoscopy could be more ill than those who underwent non-urgent endoscopy. This bias could have affected the results of our study. However, the severity index was not significantly different between the urgent and non-urgent groups in each study (Supplementary Table 2). In addition, the main outcomes, such as mortality and rebleeding rates, did not show significant differences between the two groups after propensity score matching in the two studies^5,7^. Although randomized controlled trials are recognized as the best way for overcoming this limitation, it would be difficult because of obvious ethical issues. Second, significant heterogeneity was observed in the analyses of the main outcomes. Heterogeneity was also detected in the sensitivity analyses. These data suggest that factors other than those taken into account in these analyses might have affected the outcomes. Third, some data, including rebleeding rate, other outcomes, and factors that could affect treatment outcomes including vasoactive agents and antibiotics, were unavailable in some studies, thus, weakening the interpretation of our results. Finally, we could not evaluate the publication bias because of the small number of studies included. As a rule of thumb, tests for publication bias should be used only when there are at least 10 studies included in the meta-analysis^19^.

However, this study has some strengths. This is the first meta-analysis to report the optimal timing of endoscopy in AVB. To date, current guidelines just recommend urgent endoscopy in AVB treatments. However, strong evidence is lacking except for some retrospective data about endoscopy timing. According to this study, to obtain favourable outcomes in the treatment of AVB, various factors (the patient’s condition, the feasibility of endoscopy, and the expertise of endoscopists) related to the treatment outcomes must be considered. Moreover, proper medical treatment, such as the use of vasoactive agents, prophylactic antibiotics, and fluid resuscitation, may be more important than the timing of endoscopy. If the medical condition allows, endoscopic haemostasis in AVB should not be delayed. Thus, the development of current recommendation for the appropriate endoscopy timing in patients with AVB requires further large-scale studies.

In conclusion, this meta-analysis found that endoscopy timing did not affect the mortality or rebleeding rate in patients with AVB. Although our results require careful interpretation because of the heterogeneity between the studies included, endoscopic therapy should be performed at an appropriate timing depending on each patient’s condition.

## Supplementary information

Supplementary Tables.

## Data Availability

All data are available to public and reviewers: https://osf.io/63v52/quickfiles.

## References

[1] Carbonell N (2004). Improved survival after variceal bleeding in patients with cirrhosis over the past two decades. Hepatology.

[2] D’Amico G, De Franchis R, Cooperative Study G (2003). Upper digestive bleeding in cirrhosis. Post-therapeutic outcome and prognostic indicators. Hepatology.

[3] de Franchis R, Baveno VIF (2015). Expanding consensus in portal hypertension: Report of the Baveno VI Consensus Workshop: Stratifying risk and individualizing care for portal hypertension. J. Hepatol..

[4] Garcia-Tsao G (2007). Prevention and management of gastroesophageal varices and variceal hemorrhage in cirrhosis. Hepatology.

[5] Yoo JJ (2018). Timing of upper gastrointestinal endoscopy does not influence short-term outcomes in patients with acute variceal bleeding. World J. Gastroenterol..

[6] Cheung J, Soo I, Bastiampillai R, Zhu Q, Ma M (2009). Urgent vs. non-urgent endoscopy in stable acute variceal bleeding. Am. J. Gastroenterol..

[7] Huh, C. W. *et al*. Optimal endoscopy timing according to the severity of underlying liver disease in patients with acute variceal bleeding. *Dig Liver Dis*, 10.1016/j.dld.2019.01.013 (2019). 10.1016/j.dld.2019.01.013 30803858

[8] Chen PH (2012). Delayed endoscopy increases re-bleeding and mortality in patients with hematemesis and active esophageal variceal bleeding: a cohort study. J. Hepatol..

[9] Hsu YC (2009). Delayed endoscopy as a risk factor for in-hospital mortality in cirrhotic patients with acute variceal hemorrhage. J. Gastroenterol. Hepatol..

[10] Liberati A (2009). The PRISMA statement for reporting systematic reviews and meta-analyses of studies that evaluate health care interventions: explanation and elaboration. J. Clin. Epidemiol..

[11] Kim SY (2013). Testing a tool for assessing the risk of bias for nonrandomized studies showed moderate reliability and promising validity. J. Clin. Epidemiol..

[12] Higgins JP, Thompson SG, Deeks JJ, Altman DG (2003). Measuring inconsistency in meta-analyses. BMJ.

[13] Rudler M (2018). Recalibrated MELD and hepatic encephalopathy are prognostic factors in cirrhotic patients with acute variceal bleeding. Liver Int..

[14] Thabut D (2015). Multicenter prospective validation of the Baveno IV and Baveno II/III criteria in cirrhosis patients with variceal bleeding. Hepatology.

[15] Bambha K (2008). Predictors of early re-bleeding and mortality after acute variceal haemorrhage in patients with cirrhosis. Gut.

[16] Amitrano L, Guardascione MA, Bennato R, Manguso F, Balzano A (2005). MELD score and hepatocellular carcinoma identify patients at different risk of short-term mortality among cirrhotics bleeding from esophageal varices. J. Hepatol..

[17] Moitinho E (1999). Prognostic value of early measurements of portal pressure in acute variceal bleeding. Gastroenterology.

[18] Reverter E (2014). A MELD-based model to determine risk of mortality among patients with acute variceal bleeding. Gastroenterology.

[19] Page, M. J., Higgins, J. P. T. & Sterne, J. A. C. Chapter 13: Assessing risk of bias due to missing results in a synthesis. In: Higgins, J. P. T., Thomas, J., Chandler, J., Cumpston, M., Li, T., Page, M. J., Welch, V. A. (editors). Cochrane Handbook for Systematic Reviews of Interventions version 6.0 (updated July 2019). Cochrane, Available from www.training.cochrane.org/handbook (2019).

