# Supplementary material for: Optimal endoscopy timing in patients with acute variceal bleeding: A systematic review and meta-analysis
Source: Sci Rep. 2020 Mar 4;10:4046. doi: 10.1038/s41598-020-60866-x (PMC7055310; doi:10.1038/s41598-020-60866-x)
Supplement: Supplementary file 1 — Supplementary Tables. [file 41598_2020_60866_MOESM1_ESM.pdf]

Supplementary table 1. Search terms in PubMed, Embase and Cochrane Library

PubMed	Embase	Cochrane Library
(((((((("Esophageal and Gastric Varices"[Mesh]))) OR (("Esophageal and Gastric Varices"[TW] OR "Gastric Varix"[TW] OR "Varices, Gastric"[TW] OR "Varix, Gastric"[TW] OR "Gastric Varices"[TW] OR "Esophageal Varices"[TW] OR "Esophageal Varix"[TW] OR "Varices, Esophageal"[TW] OR "Varix, Esophageal"[TW] OR "gastroesophageal varices"[TW])))) AND (((("Hemorrhage"[Mesh]) OR (("Hemorrhage"[TW] OR "Hemorrhages"[TW] OR "Bleeding"[TW]))) OR "Gastrointestinal Hemorrhage"[Mesh]) OR (("Gastrointestinal Hemorrhage"[TW] OR "Hemorrhage, Gastrointestinal"[TW] OR "Gastrointestinal Hemorrhages"[TW] OR "Hematochezia"[TW] OR "Hematochezias"[TW]))))) OR "variceal bleeding"[TW])) AND ((((((((((("Endoscopy"[Mesh]) OR (("Endoscopy"[TW] OR "Surgical Procedures, Endoscopic"[TW] OR "Procedure, Endoscopic Surgical"[TW] OR "Procedures, Endoscopic Surgical"[TW] OR "Surgical Procedure, Endoscopic"[TW] OR "Endoscopy, Surgical"[TW] OR "Surgical Endoscopy"[TW] OR "Endoscopic Surgical Procedure"[TW] OR "Endoscopic Surgical Procedures"[TW] OR "endoscopic therapy"[TW] OR "early endoscopy"[TW] OR "urgent endoscopy"[TW]))) OR "Esophagoscopy"[Mesh]) OR (("Esophagoscopy"[TW] OR "Esophagoscopies"[TW] OR "Esophagoscopic Surgical Procedures"[TW] OR "Esophagoscopic Surgical Procedure"[TW] OR "Procedure, Esophagoscopic Surgical"[TW] OR "Procedures, Esophagoscopic Surgical"[TW] OR "Surgical Procedure, Esophagoscopic"[TW] OR "Surgery, Esophagoscopic"[TW] OR "Surgical Procedures, Esophagoscopic"[TW] OR "Esophagoscopic Surgery"[TW] OR "Esophagoscopic Surgeries"[TW] OR "Surgeries, Esophagoscopic"[TW]))) OR "Gastroscopy"[Mesh]) OR (("Gastroscopy"[TW] OR "Gastroscopies"[TW] OR "Gastroscopic Surgical Procedures"[TW] OR "Gastroscopic Surgical Procedure"[TW] OR "Procedure, Gastroscopic Surgical"[TW] OR "Procedures, Gastroscopic Surgical"[TW] OR "Surgical Procedure, Gastroscopic"[TW] OR "Surgery, Gastroscopic"[TW] OR "Surgical Procedures, Gastroscopic"[TW] OR "Gastroscopic Surgery"[TW] OR "Gastroscopic Surgeries"[TW] OR "Surgeries, Gastroscopic"[TW]))) OR "Hemostasis"[Mesh]) OR (("Hemostasis"[TW] OR "Hemostases"[TW]))) OR "Hemostasis, Endoscopic"[Mesh]) OR (("Hemostasis, Endoscopic"[TW] OR "Endoscopic Hemostasis"[TW] OR "Endoscopic Hemostases"[TW] OR "Hemostases, Endoscopic"[TW]))) OR (("endoscopic band ligation"[TW] OR "endoscopic sclerotherapy"[TW]))) OR (("timing"[TW] OR "optimal timing"[TW] OR "Endoscopy Timing"[TW])))	((('esophagus varices'/exp OR ('esophageal and gastric varices' OR 'gastric varix' OR 'varices, gastric' OR 'varix, gastric' OR 'gastric varices' OR 'esophageal varices' OR 'esophageal varix' OR 'varices, esophageal' OR 'varix, esophageal' OR 'gastroesophageal varices')) AND ('bleeding'/exp OR ('hemorrhage' OR 'hemorrhages' OR 'bleeding') OR 'gastrointestinal hemorrhage'/exp OR ('gastrointestinal hemorrhage' OR 'hemorrhage, gastrointestinal' OR 'gastrointestinal hemorrhages' OR 'hematochezia' OR 'hematochezias'))) OR ('variceal bleeding' OR 'esophagus varices bleeding'/exp)) AND ('endoscopy'/exp OR ('endoscopy' OR 'surgical procedures, endoscopic' OR 'procedure, endoscopic surgical' OR 'procedures, endoscopic surgical' OR 'surgical procedure, endoscopic' OR 'endoscopy, surgical' OR 'surgical endoscopy' OR 'endoscopic surgical procedure' OR 'endoscopic surgical procedures' OR 'endoscopic therapy' OR 'early endoscopy' OR 'urgent endoscopy') OR 'esophagoscopy'/exp OR ('esophagoscopy' OR 'esophagoscopies' OR 'esophagoscopic surgical procedures' OR 'esophagoscopic surgical procedure' OR 'procedure, esophagoscopic surgical' OR 'procedures, esophagoscopic surgical' OR 'surgical procedure, esophagoscopic' OR 'surgery, esophagoscopic' OR 'surgical procedures, esophagoscopic' OR 'esophagoscopic surgery' OR 'esophagoscopic surgeries' OR 'surgeries, esophagoscopic') OR 'gastroscopy'/exp OR ('gastroscopy' OR 'gastroscopies' OR 'gastroscopic surgical procedures' OR 'gastroscopic surgical procedure' OR 'procedure, gastroscopic surgical' OR 'procedures, gastroscopic surgical' OR 'surgical procedure, gastroscopic' OR 'surgery, gastroscopic' OR 'surgical procedures, gastroscopic' OR 'gastroscopic surgery' OR 'gastroscopic surgeries' OR 'surgeries, gastroscopic') OR 'hemostasis'/exp OR ('hemostasis' OR 'hemostases') OR ('hemostasis, endoscopic' OR 'endoscopic hemostasis' OR 'endoscopic hemostases' OR 'hemostases, endoscopic') OR ('endoscopic band ligation' OR 'endoscopic sclerotherapy') OR ('timing' OR 'optimal timing' OR 'endoscopy timing'))	((([mh "Esophageal and Gastric Varices"] OR "Esophageal and Gastric Varices":ti,ab,kw OR "Gastric Varix":ti,ab,kw OR "Varices, Gastric":ti,ab,kw OR "Varix, Gastric":ti,ab,kw OR "Gastric Varices":ti,ab,kw OR "Esophageal Varices":ti,ab,kw OR "Esophageal Varix":ti,ab,kw OR "Varices, Esophageal":ti,ab,kw OR "Varix, Esophageal":ti,ab,kw OR "gastroesophageal varices":ti,ab,kw) AND ([mh "Hemorrhage"] OR "Hemorrhage":ti,ab,kw OR "Hemorrhages":ti,ab,kw OR "Bleeding":ti,ab,kw OR [mh "Gastrointestinal Hemorrhage"] OR "Gastrointestinal Hemorrhage":ti,ab,kw OR "Hemorrhage, Gastrointestinal":ti,ab,kw OR "Gastrointestinal Hemorrhages":ti,ab,kw OR "Hematochezia":ti,ab,kw OR "Hematochezias":ti,ab,kw)) OR "variceal bleeding":ti,ab,kw) AND ([mh "Endoscopy"] OR "Endoscopy":ti,ab,kw OR "Surgical Procedures, Endoscopic":ti,ab,kw OR "Procedure, Endoscopic Surgical":ti,ab,kw OR "Procedures, Endoscopic Surgical":ti,ab,kw OR "Surgical Procedure, Endoscopic":ti,ab,kw OR "Endoscopy, Surgical":ti,ab,kw OR "Surgical Endoscopy":ti,ab,kw OR "Endoscopic Surgical Procedure":ti,ab,kw OR "Endoscopic Surgical Procedures":ti,ab,kw OR "endoscopic therapy":ti,ab,kw OR "early endoscopy":ti,ab,kw OR "urgent endoscopy":ti,ab,kw OR [mh "Esophagoscopy"] OR "Esophagoscopy":ti,ab,kw OR "Esophagoscopies":ti,ab,kw OR "Esophagoscopic Surgical Procedures":ti,ab,kw OR "Esophagoscopic Surgical Procedure":ti,ab,kw OR "Procedure, Esophagoscopic Surgical":ti,ab,kw OR "Procedures, Esophagoscopic Surgical":ti,ab,kw OR "Surgical Procedure, Esophagoscopic":ti,ab,kw OR "Surgery, Esophagoscopic":ti,ab,kw OR "Surgical Procedures, Esophagoscopic":ti,ab,kw OR "Esophagoscopic Surgery":ti,ab,kw OR "Esophagoscopic Surgeries":ti,ab,kw OR "Surgeries, Esophagoscopic":ti,ab,kw OR [mh "Gastroscopy"] OR "Gastroscopy":ti,ab,kw OR "Gastroscopies":ti,ab,kw OR "Gastroscopic Surgical Procedures":ti,ab,kw OR "Gastroscopic Surgical Procedure":ti,ab,kw OR "Procedure, Gastroscopic Surgical":ti,ab,kw OR "Procedures, Gastroscopic Surgical":ti,ab,kw OR "Surgical Procedure, Gastroscopic":ti,ab,kw OR "Surgery, Gastroscopic":ti,ab,kw OR "Surgical Procedures, Gastroscopic":ti,ab,kw OR "Gastroscopic Surgery":ti,ab,kw OR "Gastroscopic Surgeries":ti,ab,kw OR "Surgeries, Gastroscopic":ti,ab,kw OR [mh "Hemostasis"] OR "Hemostasis":ti,ab,kw OR "Hemostases":ti,ab,kw OR [mh "Hemostasis, Endoscopic"] OR "Hemostasis, Endoscopic":ti,ab,kw OR "Endoscopic Hemostasis":ti,ab,kw OR "Endoscopic Hemostases":ti,ab,kw OR "Hemostases,

		Endoscopic":ti,ab,kw OR "endoscopic band ligation":ti,ab,kw OR "endoscopic sclerotherapy":ti,ab,kw OR "timing":ti,ab,kw OR "optimal timing":ti,ab,kw OR "Endoscopy Timing":ti,ab,kw)

Supplementary table 2. Patient characteristics between urgent and non-urgent groups among the five studies included

Authors	Patients (n)	Child-Pugh score (mean)	MELD score (mean)	Vital sign (Blood pressure, mmHg)	Vital sign (Heart rate, beat/min)	Prognostic score	Infection	PSM
Cheung et al.[6]	urgent: 134<br>non-urgent: 76	8.5 ± 2.0*	14.3 ± 5.3*	121 ± 16*	98 ± 20*	NA	NA	X
Hsu et al.[9]	urgent: 176<br>non-urgent: 135	NA	11.6 (8.6-14.8)**	117 (101-134)**	NA	NA	NA	X
Chen et al.[8]	urgent: 54<br>non-urgent: 47	9 (8-11)<br>9 (8-11)***	13 (9.5-18)<br>16 (11-22)***	11/37 (30%)<br>9/36 (25%)****	NA	NA	9/37 (24%)<br>15/36 (42%)***	X
Yoo et al.[5]	urgent: 173<br>non-urgent: 101	NA	15.4 ± 6.9<br>16.9 ± 9.2	116 ± 26<br>120 ± 26	96 ± 19<br>94 ± 17	9.2 ± 3.3*****<br>9.1 ± 3.9*****	NA	O
Huh et al.[7]	urgent: 317<br>non-urgent: 94	8.2 ± 2.4<br>8.5 ± 2.4	12.3 ± 7.1<br>11.5 ± 6.4	107.3 ± 25.6<br>106.2 ± 22.1	98.4 ± 21.0<br>88.7 ± 18.5	4.0 ± 1.1*******<br>4.0 ± 0.9*******	54 (17.0%)<br>9 (9.6%)	O

MELD, model for end-stage liver disease; NA, not available; PSM, propensity score matching

* Only total mean value was available. They described that there was no difference between urgent and non-urgent groups.

** Data expressed as median. Only total median value was available.

*** Data expressed as median. Data available only patients with hematemesis.

**** Shock at arrival. Data available only patients with hematemesis.

***** Glasgow-Blatchford score

****** Charlson comorbidity score

Supplementary table 3. Other characteristics of the studies included in the meta-analysis

Authors	Mortality definition	Cause of death	Rebleeding definition	Criteria to perform urgent endoscopy	Antibiotics use	Vasoactive agent	Type of variceal bleeding (OV/GV/Both)	Method for haemostasis (EBL/EIS)	Transfusion policy
Cheung et al.[6]	In hospital mortality	Available*	Available	NA	Yes**	Octreotide	191/12/7	171/19	NA
Hsu et al.[9]	In hospital mortality	NA	NA	Available	NA	Terlipressin: 124<br>Somatostatin: 179<br>None: 8	191/120	121/155/3	NA
Chen et al.[8]	6week mortality	Available***	Available	Available	Yes**	Somatostatin	80/21	NA	NA****
Yoo et al.[5]	6week mortality	NA	NA	Available	Yes**	Terlipressin	NA	NA	NA
Huh et al.[7]	6week mortality	NA	Available	Available	Ceftriaxone 1-2g/day, 5-7days	Terlipressin	339/72	NA*****	NA

EBL, endoscopic band ligation; EIS; endoscopic injection sclerotherapy; GV, gastric varix; NA, not available; OV, oesophageal varix.

* Multiorgan failure owing to sepsis (n = 7), respiratory failure (n = 3), liver failure (n = 3), hemorrhagic shock (n = 2), recurrent variceal bleeding (n = 1), cardiac arrest (n = 1), anoxic brain injury (n = 1), intracerebral hemorrhage (n = 1), and unknown causes (n = 1).

** Type and dose of antibiotics were not available.

*** Fourteen patients (31%) died due to hepatic failure.

****Packed red blood cell was given to maintain hemoglobin of 8 g/dl.

*****Oesophageal varices and type 1 gastroesophageal varices were treated with EBL, and type 2 gastroesophageal varices and isolated gastric varices were treated with sclerotherapy.

Supplementary table 4. Results of sensitivity analyses excluding studies that did not compare baseline characteristics between the urgent and non-urgent groups

Sensitivity analyses	Event rate (95% CI)	Heterogeneity
**Mortality**		
Sensitivity analysis excluding studies that did not compare baseline characteristics between the two groups	1.19 (95% CI=0.59-2.43)	p = 0.09, I^2 = 58%
**Rebleeding**		
Sensitivity analysis excluding excluding the study that did not compare baseline between the two groups	1.30 (95% CI=0.71-2.36)	p = 0.07, I^2 = 63%
